# DNA damage strength modulates a bimodal switch of p53 dynamics for cell-fate control

**DOI:** 10.1186/1741-7007-11-73

**Published:** 2013-06-21

**Authors:** Xi Chen, Jia Chen, Siting Gan, Huaji Guan, Yuan Zhou, Qi Ouyang, Jue Shi

**Affiliations:** 1 Center for Quantitative Systems Biology and Department of Physics, Hong Kong Baptist University, 224 Waterloo Road, Kowloon Tong, Kowloon, Hong Kong, China; 2 Center for Quantitative Biology and State Key Laboratory for Mesoscopic Physics, Peking University, Beijing, 100871, P. R. China

## Abstract

**Background:**

The p53 pathway is differentially activated in response to distinct DNA damage, leading to alternative phenotypic outcomes in mammalian cells. Recent evidence suggests that p53 expression dynamics play an important role in the differential regulation of cell fate, but questions remain as to how p53 dynamics and the subsequent cellular response are modulated by variable DNA damage.

**Results:**

We identified a novel, bimodal switch of p53 dynamics modulated by DNA-damage strength that is crucial for cell-fate control. After low DNA damage, p53 underwent periodic pulsing and cells entered cell-cycle arrest. After high DNA damage, p53 underwent a strong monotonic increase and cells activated apoptosis. We found that the damage dose-dependent bimodal switch was due to differential Mdm2 upregulation, which controlled the alternative cell fates mainly by modulating the induction level and pro-apoptotic activities of p53.

**Conclusions:**

Our findings not only uncover a new mode of regulation for p53 dynamics and cell fate, but also suggest that p53 oscillation may function as a suppressor, maintaining a low level of p53 induction and pro-apoptotic activities so as to render cell-cycle arrest that allows damage repair.

## Background

An efficient and precisely controlled DNA-damage response is crucial for cell survival. In mammalian cells, the p53 regulatory pathway, consisting mainly of the tumor suppressor protein p53 and its downstream transcriptional targets, plays an essential role in mediating this crucial stress response to chromosomal damage [1-3]. Depending on the type and severity of DNA damage, the p53 pathway activates either cell-cycle arrest that allows repair of the damage or alternatively, death of the cell through apoptosis, but the mechanism by which variation in DNA-damage strength differentially regulates p53 pathway dynamics, and the effect that this has on cell fate, are poorly understood.

Mild DNA damage generally induces a moderate increase in p53 level, and results in transient cell-cycle arrest that allows damage repair, whereas severe and possibly irreparable DNA damage leads to a large increase in p53, followed by cell death [4]. DNA damage is known to upregulate p53 by attenuating its interaction with Mdm2, the E3 ubiquitin ligase that targets p53 for proteasome-mediated degradation [5,6]. Less clear is how the severity of DNA damage modulates the degree of attenuation in p53-Mdm2 interactions, giving rise to differential p53 expression that results in these alternative cell fates. Also not clear is how the dynamics of p53 differ with variation in damage level, and what role any changes in the dynamics might play in the downstream function of p53. Most previous studies of p53 dynamics have focused only on the response to transient DNA damage induced by gamma or UV irradiation, which has been shown to induce an intriguing oscillatory behavior of p53, which is largely independent of DNA damage level [7-11]. Moreover, although recent results, including our own [11,12], have shown that p53 dynamics can be altered by using nutlin to inhibit Mdm2, the observed change in p53 dynamics was again independent of damage severity.

To investigate whether and how DNA-damage strength modulates p53 dynamics and subsequently alters cell-fate outcome, we performed a dose–response study by measuring the real-time p53 dynamics under variable DNA damage generated by etoposide, a chemotherapeutic compound that induces sustained DNA damage. Using time-lapse microscopy, we quantified p53 dynamics at the single-cell level, and correlated the dynamics with cell fates. Our results showed that p53 dynamics exhibit ‘switch-like’ behavior, changing from oscillatory dynamics at low damage to monotonic increase at high damage. Moreover, this damage dose-dependent, bimodal switch of p53 dynamics was found to regulate cell fate mainly by modulating the p53 induction level and its pro-apoptotic activities as a function of DNA-damage strength. Our results suggest that under certain conditions, p53 oscillations may act as a mechanism to suppress p53 induction, thereby restraining its pro-apoptotic activities and promoting cell-cycle arrest that allows DNA-damage repair.

## Results

### Dynamics of nuclear p53 is damage dose-dependent

**Figure 1:**
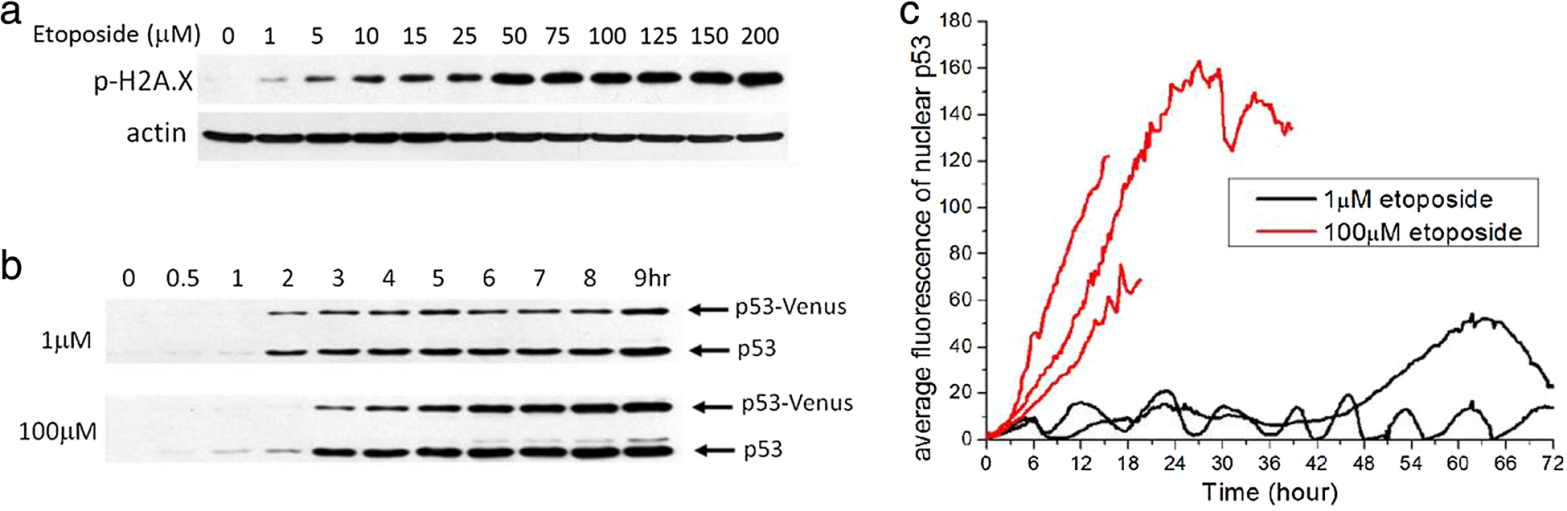
**Differential p53 dynamics as a function of DNA-damage strength. (a)** Phospho-H2A.X levels (DNA damage marker) in U-2 OS cells treated with increasing doses of etoposide for 12 hours. Actin served as a loading control. **(b)** Induction kinetics of p53-Venus and endogenous p53 in the clonal U-2 OS report line treated with 1 and 100 μmol/l etoposide at the indicated time points (in hour). The upper band is p53-Venus (around 80 kDa) and the lower band is endogenous p53 (approximately 53 kDa). **(c)** Single-cell trajectories of p53 dynamics at the nucleus quantified from p53-Venus fluorescence. U-2 OS cells were treated with either 1 μmol/l (black lines) or 100 μmol/l (red lines) of etoposide at time 0. Individual cells were tracked for 72 hours or until cell death occurred. The abrupt end of p53 trajectories before 72 hours corresponds to the time of death.

To obtain data on p53 dynamics and cell fate as a function of DNA-damage strength, we performed dose titration of a DNA-damaging drug, etoposide, on U-2 OS cells expressing a fluorescent p53 reporter. The reporter cell line was generated by infecting U-2 OS cells with lentivirus encoding an established wild-type p53-Venus reporter construct [9]. For the study, we selected an isogenic clone that exhibited behaviors most similar to the parental line (refer to discussion below). We used etoposide as the DNA-damage stimulus, as it is known to trigger DNA double-strand breaks (DSBs) by inhibiting topoisomerase II [13,14]. To select the appropriate range of etoposide dosage for activating variable amounts of DNA damage, we first measured the level of DNA damage under different etoposide doses. DNA damage, as indicated by the DNA-damage marker, phospho-H2A.X, was present after 12 hours of drug treatment (Figure 1a), and the degree of DNA damage increased with increasing dosage of etoposide from 1 μmol/l to 75 to 100 μmol/l.

To verify that dynamics of the p53-Venus reporter construct in our clonal reporter line mimicked the endogenous p53, we compared the dynamics of the endogenous and fluorescently tagged p53 by western blotting. The dynamics of the p53-Venus construct that we used have been extensively studied previously in MCF7 cells, which all showed that the reporter construct behaved similarly to the endogenous p53 [9,10]. Our data from the U-2 OS clone also showed similar induction dynamics between p53-Venus and its endogenous counterpart (Figure 1b; note that the level of p53-Venus reporter is about half of the endogenous p53. For quantified results of western blots, see Additional file 1: Figure S1), when cells were treated with both low and high doses of etoposide. Therefore, the fluorescent p53 reporter is probably regulated similarly to the endogenous wild-type p53, and can be used to infer p53 dynamics in response to DNA damage.

From time-lapse microscopy movies, we quantified the real-time induction dynamics of p53 by measuring average Venus fluorescence in the nucleus and cytoplasm, respectively. In general, the fluorescent signal of p53-Venus in the nucleus was significantly higher than that in the cytoplasm. Single-cell p53 fluorescence trajectories showed two distinctive dynamic features: 1) nuclear p53 exhibited periodic pulsing at low drug dose, but monotonic increase at high dose; and 2) the amplitude of monotonically elevated p53 was substantially higher than that of the pulsing mode (Figure 1c; see Additional file 1: Figure S2). These bimodal, damage dose-dependent p53 dynamics are in stark contrast to the dose-independent oscillatory response of p53 to gamma irradiation previously identified in single mammalian cells [8]. The observation of a switch from oscillatory dynamics to a monotonic increase in p53 as DNA damage increased also suggests an intriguing new transitional regulation of p53.

### The distinct mode of p53 dynamics strongly correlates with cell fate

**Figure 2:**
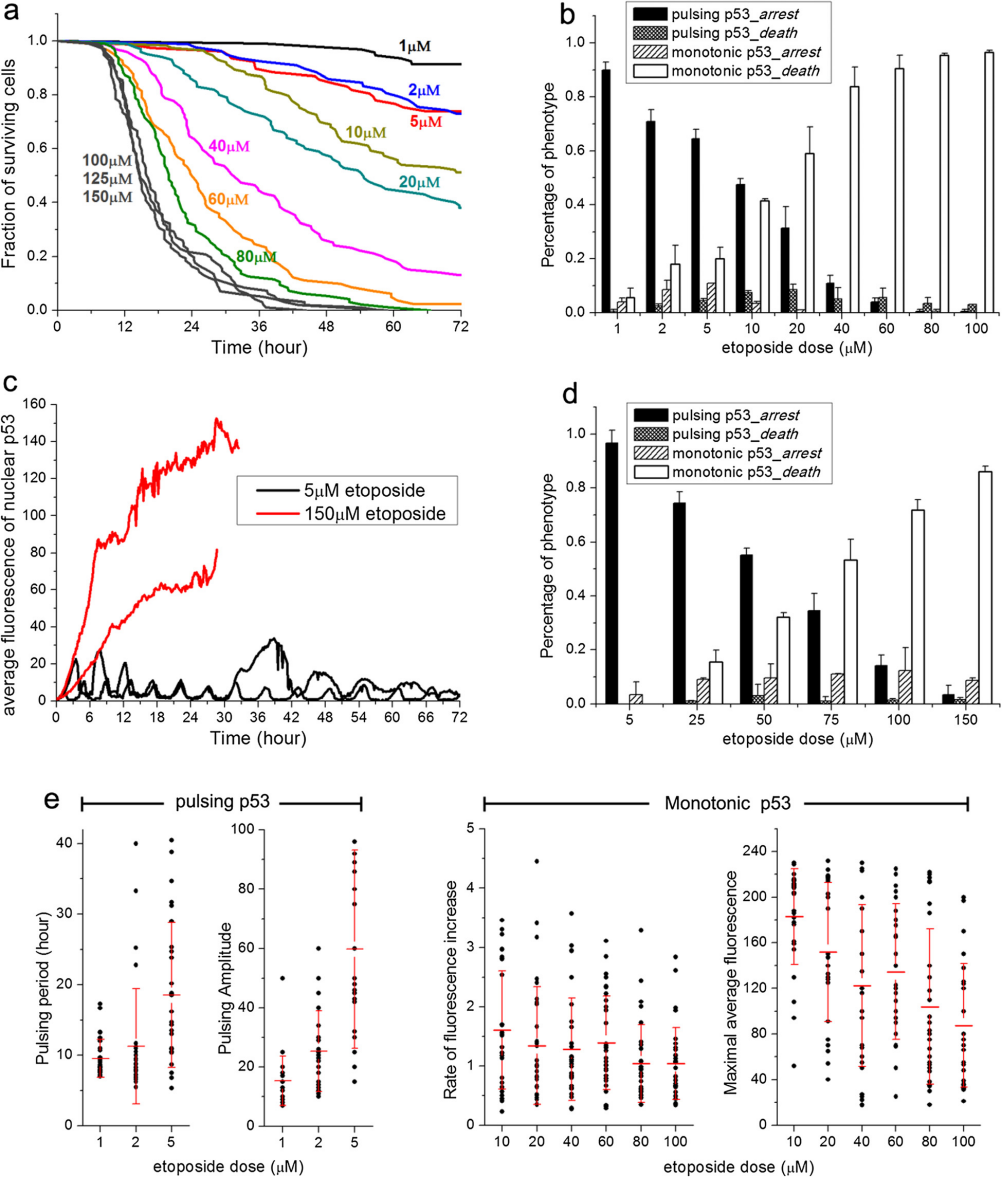
**Bimodal switch of p53 dynamics and its correlation with cell-fate outcome. (a)** Cumulative survival curves for U-2 OS cells treated with etoposide at the indicated dose from 1 to 150 μmol/l. The total number of cells analyzed for each curve ranged from 77 to 113, and varied between conditions. Individual cells were monitored by phase-contrast and fluorescence time-lapse microscopy from drug addition for 72 hours or until morphological death occurred. Kinetics of cell death were plotted as cumulative survival curves. **(b)** Distribution of the four response phenotypes with respect to p53 dynamics and cell fate (as discussed in the text) to DNA damage triggered by etoposide at the indicated doses. Error bars show standard deviations (SDs) of data from two independent experiments. **(c)** Single-cell trajectories of p53 dynamics at the nucleus quantified from the A549-p53 reporter line. **(d)** Distribution of the four response phenotypes in A549 cells at the indicated etoposide doses. The total number of cells analyzed for each concentration ranged from 81 to 106. Error bars show SDs of data from two independent experiments. **(e)** Characteristics of the two p53 dynamic modes in individual cells (denoted by the black dots) and the population average (denoted by the red bars). (Left) pulsing period and pulsing amplitude; (right) rate of monotonic increase and maximum amplitude. Thirty cells were analyzed for each drug concentration. Error bars show SDs of data from the 30 individual cells.

Besides p53 dynamics, cumulative survival curves (quantified by analyzing individual cell fate from the time-lapse movies) showed that cell fate was also altered. As the dose of etoposide increased from 1 to 100 μmol/l, the cell-fate profile shifted from primarily cell-cycle arrest (scored by absence of cell division in 72 hours) to primarily cell death, with 100 μmol/l being the saturating dose for the cell-death response (Figure 2a). The dose response of the U-2 OS p53 reporter line was similar to that of the parental line, with the reporter line being slightly more sensitive to etoposide, probably due to the extra copies of p53 (see Additional file 1: Figure S3). Moreover, the single-cell data from the reporter line showed that the phenotypic outcome of arrest versus death correlated strongly with the dynamic mode of p53, as overall 86% of the cells (summed over all drug concentrations) that showed p53 pulsing went into cell-cycle arrest, and more than 96% of those with monotonic p53 induction died. Figure 2b shows the distributions of the four observed response phenotypes at different etoposide doses: 1) pulsing p53 followed by cell-cycle arrest, 2) pulsing p53 followed by cell death, 3) monotonic increase of p53 followed by cell-cycle arrest, and 4) monotonic increase of p53 followed by cell death. At all etoposide doses, phenotypes 1 and 4 accounted for more than 85% of the cells seen. In particular, at low DNA damage (etoposide ≤5 μmol/l), more than 65% of cells showed periodic pulsing and cell-cycle arrest, whereas at high DNA damage (etoposide ≥80 μmol/l) more than 95% cells died after monotonic elevation of p53.

To determine whether other mammalian cells exhibit a bimodal switch of p53 dynamics that correlates with cell fate as a function of DNA-damage strength, we generated another clonal fluorescent p53 reporter cell line in A549 cells. Dose titration results of the A549-p53 reporter line showed that A549 was more resistant than U-2 OS to etoposide, requiring higher concentrations to induce both cell-cycle arrest (5 μmol/l) and cell death (150 μmol/l) to near saturation level (see Additional file 1: Figure S4). In A549 cells, p53 again showed a bimodal switch of dynamics similar to that observed in U-2 OS cells, namely, pulsing at low DNA damage and monotonic increase at high DNA damage (Figure 2c). The strong correlation between cell fate and the two distinct p53 dynamic modes was also seen in A549 cells (Figure 2d). Overall, 97% of A549 cells that displayed p53 pulsing went into cell-cycle arrest, and more than 83% of those with monotonic p53 induction died. Our data thus suggest that the bimodal switch and its correlation with alternative cell fate are common in at least some mammalian cells in response to variable DNA damage.

We noted that although the correlation between cell fate and p53 induction dynamics was evident across the entire cell population, there was strong cell-to-cell variation in terms of the quantitative characteristics. We quantified the single-cell data and population average for the two p53 dynamic modes observed in U-2 OS cells at different etoposide concentrations (Figure 2e). For the pulsing mode, the pulsing period of the initial two to five pulses varied between individual cells from approximately 6 to approximately 40 hours, and the pulsing amplitude spanned a nearly eight-fold range. Furthermore, more than 50% of cells displayed irregular oscillatory behaviors with change in either pulsing period or amplitude over time. The strong single-cell variability was also evident for the monotonic induction mode, as shown by the large standard deviations in the population averages in terms of both the rate of p53 increase and the maximal p53 level (Figure 2e). In addition, our results appeared to show distinct dose dependence of the two p53 dynamic modes. Both the average pulsing period and the pulsing amplitude exhibited a moderate increase as the etoposide concentration increased, whereas the rate of increase and the maximal p53 level under the monotonic induction mode was largely dose-independent.

### Mdm2 expression level controls the dynamic mode of p53 induction

**Figure 3:**
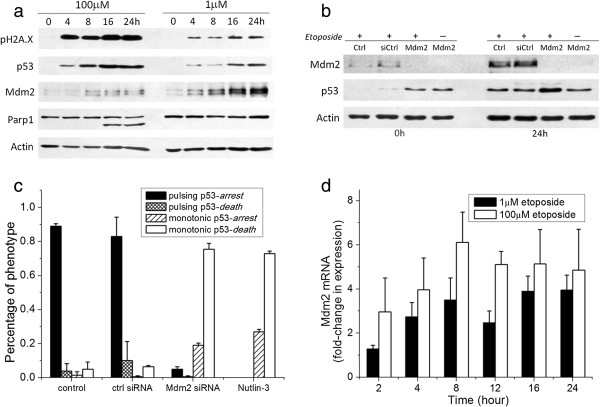
**Regulation of bimodal p53 dynamics by Mdm2 expression. (a)** Comparison of kinetics of selected proteins/protein modifications by western blot analysis for U-2 OS cells treated with 100 and 1 μmol/l etoposide, respectively. Actin served as a loading control. **(b)** Mdm2 and p53 expression levels with the indicated RNA interference (RNAi) treatment after 0 and 24 hours of 1 μmol/l etoposide treatment. +: With etoposide; −: without etoposide. **(c)** Distribution of the four response phenotypes for the indicated treatments: control (no small interfering (si)RNA), control siRNA, Mdm2 siRNA, and 10 μmol/l nutlin-3, in combination with 1 μmol/l etoposide in U-2 OS cells. The total number of cells analyzed for each treatment ranged from 69 to 99. Error bars show standard deviations of data from two independent experiments. **(d)** Real-time quantitative (q)PCR analysis of Mdm2 transcript level at 1 μmol/l (filled column) and 100 μmol/l etoposide (empty columns). Error bars show standard deviations of data from three independent experiments.

p53 level is known to be negatively regulated by the E3 ubiquitin ligase Mdm2, while at the same time Mdm2 is transcriptionally activated by p53. Such a negative feedback loop between p53 and Mdm2 maintains p53 at a low level in unstressed cells. Upon induction of DNA damage, the inhibitory interaction between p53 and Mdm2 is disrupted by phosphorylation of both proteins, resulting in p53 accumulation [15]. To test whether the bimodal switch of p53 induction is a result of differences in Mdm2 activation, we performed immunoblotting to compare the dynamics of ensemble p53 and Mdm2 at 1 and 100 μmol/l etoposide, as under these two extreme concentrations, cells largely exhibited the same p53 dynamics of either pulsing or monotonic increase. The level of p53 increase was substantially higher at high drug dose than low dose for U-2 OS cells (Figure 3), agreeing with the live-cell imaging observations. Intriguingly, whereas expression of Mdm2 steadily increased at 1 μmol/l etoposide, its expression was largely suppressed at 100 μmol/l. Our data thus indicate that attenuation in upregulation of Mdm2 at high DNA damage may enable p53 to accumulate continuously to a high level, exhibiting monotonic elevation that is fundamentally different from the pulsing mode triggered by low damage.

To further confirm the role of Mdm2 in regulating the bimodal p53 dynamics, we attenuated Mdm2 activity in U-2 OS cells by using either RNA interference (RNAi) knockdown or nutlin-3, a small-molecule inhibitor of Mdm2 [16], and compared the resulting p53 dynamics and cell fate with those after treatment of 1 μmol/l etoposide alone (low DNA damage). Mdm2 knockdown efficiencies were generally greater than 90%, and Mdm2 knockdown alone (without etoposide) increased p53 to a level similar to that seen with 1 μmol/l etoposide alone (Figure 3b). Approximately 70% of U-2 OS cells under Mdm2 knockdown alone continued to divide, and less than 10% cell death occurred in the 72 hours of the imaging experiment. Figure 3c compares the distributions of the four different phenotypes, as discussed above, after treatment with 1 μmol/l etoposide plus one of the following four conditions: control (no siRNA), control siRNA (control for the pro-apoptotic effect of transfection), Mdm2 knockdown, and 10 μmol/l nutlin-3. We chose to use 10 μmol/l nutlin-3 because at this concentration nutlin-3 alone induced little cell death (<4% of U-2 OS cells died after 72 hours). Control siRNA transfection slightly sensitized cells to etoposide-induced cell death (approximately 5%), but did not alter distribution of the phenotypic responses, with about 90% of cells exhibiting pulsing p53 followed by cell-cycle arrest, similar to that of control cells. By contrast, Mdm2 knockdown not only significantly upregulated p53 level (Figure 3b) and changed the mode of p53 dynamics from being approximately 90% oscillatory to approximately 95% monotonic increase, but also shifted cell fate from approximately 90% cell-cycle arrest to approximately 80% cell death (Figure 3c). Similar changes in p53 dynamics and cell fate were also seen with etoposide plus nutlin-3. These data strongly suggest that the bimodal p53 dynamics are generated by differential Mdm2 upregulation modulated by DNA-damage strength, and that the bimodal switch functions as a crucial control over arrest versus death, as cell fate can be altered by changing p53 dynamics.

Attenuation of Mdm2 expression at high DNA damage could be due to reduced gene transcription or translation, or increased protein degradation. Real-time quantitative PCR (qPCR) results of Mdm2 showed that the gene-expression level of Mdm2 was largely similar under low and high etoposide concentrations (Figure 3d). Therefore, the distinct Mdm2 expression that we observed at the protein level in response to variable DNA-damage strength is not due to differential gene expression, but possibly due to differences in translation or Mdm2 protein degradation, such as by HAUSP [17] and ATM [18].

### Bimodal switch of p53 dynamics modulates the overall transcriptional activity of p53

**Figure 4:**
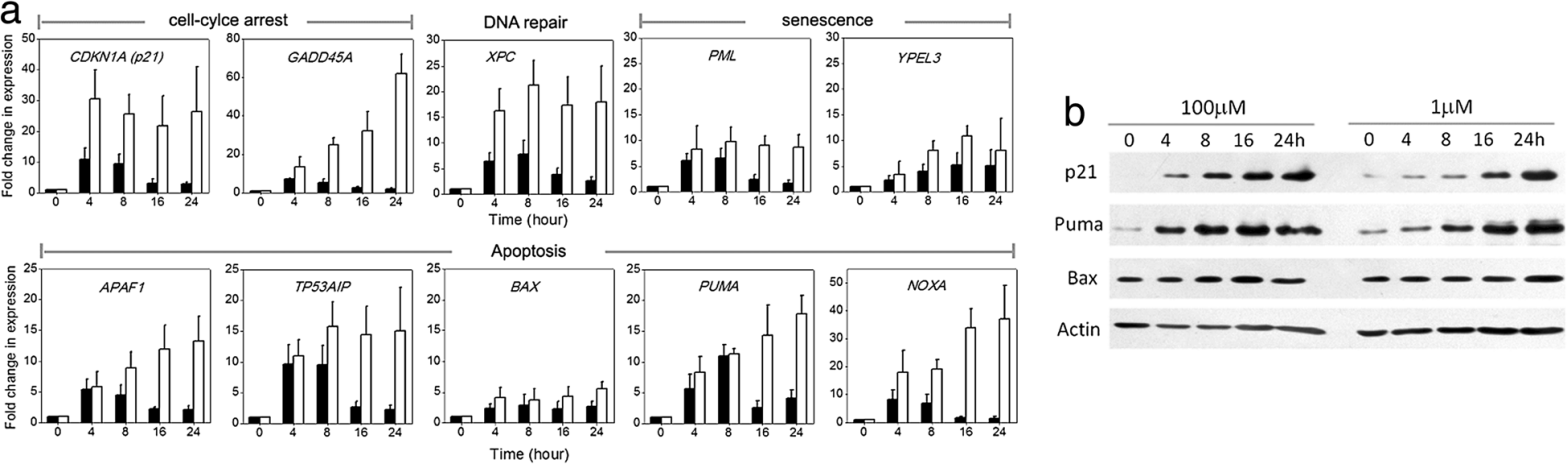
**p53 transcriptional activity and its effect on cell fate. (a)** Gene expression of a set of well-known p53 target genes by real-time quantitative (q)PCR analysis in U-2 OS cells treated with 1 μmol/l (filled columns) and 100 μmol/l (empty columns) etoposide, respectively. Error bars show standard deviations of data from three independent experiments. **(b)** Comparison of expression of selected p53 target genes by western blotting analysis.

p53 is known to modulate cell fate through both its transcriptional activity in the nucleus, which upregulates downstream target genes, and its transcription-independent pro-apoptotic activity in the cytoplasm [4,19]. To determine whether the bimodal switch of p53 dynamics exerts control over alternative cell fates by modulating the transcriptional activity of p53 in upregulating distinct pro-survival and pro-death genes, we measured, by real-time qPCR, the gene-expression dynamics of a set of well-characterized p53 target genes that are known to regulate cellular response to DNA damage [20]. These target genes include those involved in cell-cycle arrest (*CDKN1A* (p21) and *GADD45A*), DNA repair (*XPC*), senescence (*PML* and *YPEL3*) [21,22] and apoptosis (*APAF1*, *TP53AIP1*, *BAX*, *PUM*A and *NOXA*). All target genes that we profiled, except BAX (largely not induced), showed much higher upregulation in response to high DNA damage (Figure 4a), when p53 was strongly elevated in the monotonic mode, irrespective of their particular functions. Of the three most highly induced genes under monotonic p53 in response to high DNA damage were two cell-cycle arrest genes, that is, *CDKN1A* (approximately 30-fold increase) and *GADD45A* (approximately 60-fold), and one pro-death gene, *NOXA* (approximately 35-fold).

To further confirm the similar induction dynamics of pro-survival and pro-death genes at the protein level, we compared, by western blotting, expression levels of p21 (CDKN1A), PUMA, and BAX from U-2 OS cells treated with 1 μmol/l (low damage) and 100 μmol/l (high damage) etoposide. Expression of both the pro-survival gene p21 and the pro-death gene PUMA were significantly induced under both low and high DNA-damage levels, whereas expression of another important pro-death gene, BAX, was not upregulated (Figure 4b, e). These largely agreed with the qPCR data. Hence, our results suggest that the bimodal switch of p53 dynamics modulates the transcriptional activation of pro-survival and pro-death genes similarly, and do not differentially activate these distinct genes in a damage dose-dependent manner. The observed bimodal switch did not appear to regulate cell fate through the previously reported mechanisms of differential transactivation of pro-survival and pro-apoptotic promoters [23], or through the p53-mediated transrepression of pro-survival genes [24]. Instead, our data point to a mechanism in which pulsing p53 retains a low level of p53 with target gene expression sufficient only for inhibiting cell-cycle progression, whereas the monotonic p53 results in strong induction of p53 that activates sufficiently high levels of pro-death genes to trigger cell death, superseding the concomitant cell-cycle arrest response.

### Bimodal switch of p53 dynamics modulates the p53 cytoplasmic localization, and this correlates with the pro-apoptotic activity

**Figure 5:**
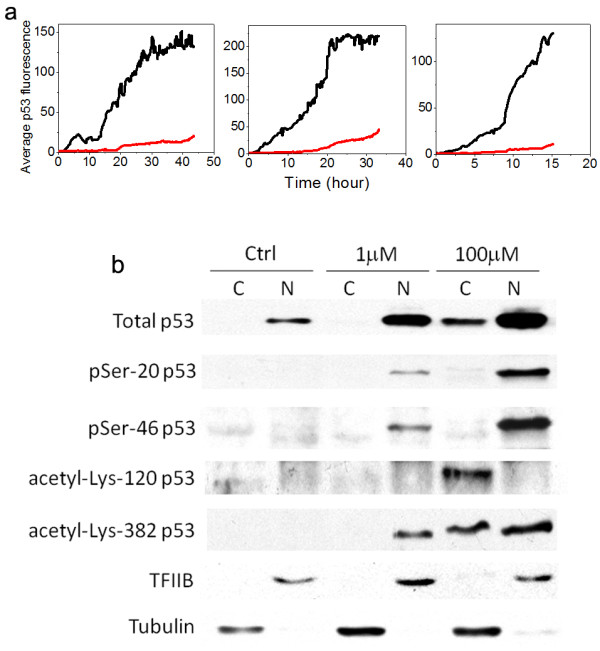
**Bimodal switch of p53 dynamics modulates the p53 cytoplasmic localization. (a)** Representative single-cell trajectories of average p53 fluorescence in the nucleus (black line) and cytoplasm (red line) under 100 μmol/l etoposide. **(b)** Western blotting analysis of p53 levels and the indicated p53 post-translational modifications in the nucleus and cytoplasm of U-2 OS cells treated with no drug, 1 μmol/l etoposide (treated for 24 hours) and 100 μmol/l etoposide (treated for 12 hours), respectively. To confirm the quality of the subcellular fractionation, tubulin was used as a cytoplasmic marker and transcriptional factor II B (TFIIB) as a nuclear marker.

The single-cell imaging data showed that although under low etoposide dosage the cytoplasmic p53 signal was close to that of the fluorescence background, there was an observable increase in cytoplasmic p53 fluorescence in response to high drug dosage, although the majority of the activated p53 was localized in the nucleus under both conditions. Shown in Figure 5a are representative single-cell trajectories of p53 fluorescence in the cytoplasm, relative to that in the nucleus from U-2 OS cells treated with 100 μmol/l etoposide. The average p53 fluorescence in the cytoplasm was found to be about 10% of that in the nucleus. Cell-to-cell variability was again evident, with the abundance of cytoplasmic p53 ranging from about 5% to 25% of that in the nucleus. The single-cell data indicated that the cytoplasmic accumulation of p53, which may be proportional to the cytoplasmic pro-apoptotic activity of p53, was modulated by the bimodal switch of p53 dynamics as a function of DNA-damage strength.

To determine the level of cytoplasmic p53 at the ensemble level, we performed subcellular fractionation. Using western blotting, we compared levels of nuclear and cytoplasmic p53 in U-2 OS cells treated with no drug, 1 μmol/l and 100 μmol/l etoposide, respectively (Figure 5b). Tubulin and transcriptional factor II B (TFIIB) were used as cytoplasmic and nuclear markers to confirm the quality of fractionation. In contrast to cells treated with 1 μmol/l etoposide, in which little cytoplasmic p53 was detected, treatment with 100 μmol/l etoposide induced substantial accumulation of cytoplasmic p53 (approximately 15% of that in the nucleus). The level of cytoplasmic p53 correlated with the differential cell fate, that is, more than 90% cell-cycle arrest at low damage, where cytoplasmic p53 was low, and more than 95% cell death at high damage, where cytoplasmic p53 was significant. These data thus suggest that in addition to the transcriptional activation of pro-death genes, bimodal switching of p53 dynamics may also regulate cell fate by modulating cytoplasmic accumulation of p53, which possibly correlates with its pro-apoptotic activity in the cytoplasm, as DNA damage increases.

Besides the induction level, activities of p53 are also known to be regulated extensively by post-translational modifications, such as phosphorylation and acetylation [25,26]. Previous studies have identified several post-translational modifications on p53 that play crucial roles in regulating differential cell fate, such as Ser-20 phosphorylation by Chk1/Chk2 [27], Ser-46 phosphorylation by HIPK2 [28], Lys-120 acetylation by Tip60 [29], and Lys-382 acetylation by CBP/p300 [30]. To determine whether these modifications affect the cytoplasmic accumulation of p53, we measured, by western blotting, the above four post-translational modifications in the cytoplasmic and nuclear fractions of p53, respectively (Figure 5b). Whereas the extent of p53 phosphorylation at serine-20 and serine-46 was largely proportional to the expression level of p53, irrespective of the sub-celluar localization, the cytoplasmic fraction of p53 appeared to be preferentially modified by acetylation at lysine-120 and lysine-382, indicating that these two post-translational modifications may potentially have important roles in regulating p53 translocation to cytoplasm and/or its cytoplasmic pro-apoptotic activity.

## Discussion

Our observations that p53 dynamics can switch modes in a damage dose-dependent manner, and that cell fate can be altered by changing the dynamic mode of p53, reveal a new mechanism by which the p53 pathway regulates differential DNA-damage responses. By examining both the transcriptional and non-transcriptional functions of p53 at distinct damage levels, we further determined that the bimodal switch of p53 dynamics regulates differential cell fate, mainly by modulating the p53 induction level and pro-apoptotic activities. Under moderate DNA damage, p53 undergoes periodic pulsing, which might act to retain a low level of p53 induction with low induction of pro-death genes and low cytoplasmic accumulation of p53. In this low damage mode, the p53 pro-apoptotic activities are suppressed and cell-cycle arrest dominates. In contrast, a high level of DNA damage triggers strong monotonic elevation of p53, which activates high levels of pro-death genes and also promotes the cytoplasmic accumulation of p53. In this high damage mode, the overall pro-apoptotic activities of p53 increase, resulting in cell death and superseding the cell-cycle arrest response. Our data not only point to the bimodal switch of p53 dynamics as a crucial control over cell fate in response to variable DNA damage, but also suggest that p53 oscillations probably function as a suppressor through maintaining a low induction level of p53, which suppresses its pro-apoptotic activities and renders cell-cycle arrest that allows repair at the time of low DNA damage.

The fact that p53 fundamentally changes its dynamic mode, that is, switching from pulsing at low damage to monotonic increase at high damage, poses important new questions regarding the transitional behavior and regulation of p53 pathway dynamics. For instance, what is the crucial damage intensity that triggers the change in p53 dynamics? Which p53 pathway components control this crucial damage threshold? Is the bimodal transition sharp or gradual as a function of damage level? Answering these questions will probably require analysis of the larger p53 pathway beyond the p53-Mdm2 negative feedback loop, so as to obtain knowledge of other feedback structures in the p53 regulatory pathway, such as those involved in damage sensing, p53 upregulation, and modification. By perturbing key feedback components connected to the central p53-Mdm2 loop and examining how the threshold, quantitative characteristics, and single-cell variability of the p53 bimodal switch are altered, a more quantitative understanding of the bimodal switch will be acquired. To identify the regulatory dependence of the bimodal switch on particular pathway motifs, investigations that combine experimental measurements and theoretical analysis of the pathway dynamics are clearly needed. We believe results from such further investigations will provide important insights into novel strategies of signaling-pathway control that may be common in mammalian cells.

Although our results showed that differential Mdm2 upregulation was responsible for altering p53 dynamics, the exact mechanism by which Mdm2 is differentially regulated has not been determined. We found that attenuation of Mdm2 expression at high DNA damage was not due to reduction in gene transcription, suggesting that reduction in translation or increased protein degradation may be the potential molecular regulators. Work by Stommel and Wahl [18] showed that auto-degradation of Mdm2 is regulated by DNA-damage kinases, such as ATM. This suggests one possible mechanism for the observed attenuation in Mdm2 in response to high DNA damage, is that as DNA damage increases, further elevation of ATM activity triggers additional phosphorylation of Mdm2, promoting the auto-degradation of Mdm2 and thus reducing its protein level. Further study will be required to determine the detailed involvement of increased Mdm2 degradation in light of the bimodal regulation of p53. Moreover, we identified differential post-translational modifications of p53 in the nucleus and cytoplasm as a function of DNA-damage dose, in particular for acetylation of p53 at lysine-120 and lysine-382. However, important questions remain, which require further, more detailed mechanistic study, regarding whether and how these two, or additional, post-translational modifications regulatep53 control over cell fate through modulating its cytoplasmic localization and/or its cytoplasmic pro-apoptotic activity.

Our study has shed new light on how the dual functions of p53 are modulated as a function of DNA-damage strength and differentially contribute to the alternative phenotypic outcome, as we found alteration in p53 dynamics mainly affects the p53 induction level and its pro-apoptotic activities in a damage dose-dependent manner. Although the decision between cell-cycle arrest and cell death mediated by the bimodal p53 dynamics was mainly determined by the level of p53 pro-apoptotic activities for the system that we studied, it is likely that the p53 pathway-mediated DNA-damage responses vary depending on the DNA-damage stimulus and the cell type investigated. Therefore, single-cell p53 studies for additional damage stimuli and cell types are needed to examine potential variability in both the bimodal switch and its regulatory effect on the dual functions of p53.

## Conclusions

In this study, using highly quantitative single-cell microscopy assays, we elucidated a novel mechanism by which DNA damage activates a bimodal switch of p53 dynamics in a damage dose-dependent manner, subsequently regulating alternative phenotypic outcome of cell-cycle arrest and cell death. Our findings not only provide important new insights into understanding the differential DNA-damage response mediated by p53, but also produce quantitative new data for exploring the transitional behavior and regulation of p53 pathway dynamics for cell-fate control.

## Methods

### p53-Venus reporter cell line

The U-2 OS and A549 cell lines were purchased from American Type Culture Collection (ATCC, Manassas, VA, USA) and cultured at 37°C with 5% CO_2_ in McCoy’s 5A or F-12K medium supplemented with 10% fetal calf serum (FCS), 100 U/ml penicillin and 100 μg/ml streptomycin. To generate fluorescent reporter cell for live-cell imaging of p53 dynamics, we infected U-2 OS and A549 cells with lentiviruses encoding an established p53-Venus reporter construct (consisting of wild-type p53 fused to a yellow fluorescent protein, Venus; generous gift from Dr Galit Lahav, Department of Systems Biology, Harvard Medical School, USA) [9], and selected isogenic clones.

### Chemicals and reagents

Etoposide was purchased from Sigma-Aldrich (St. Louis, MO, USA). siRNA for knocking down Mdm2 (#4457298) was purchased from Ambion Inc. (Austin, TX, USA), and the Mdm2 siRNA was used at a final concentration of 20 nmol/l for RNAi in all experiments. The non-targeting siRNA control was On-Target plus siControl (#D-001810-01; Dharmacon Inc., Lafayette, CO, USA). All siRNA transfections were performed using HiPerFect (Qiagen Inc., Valencia, CA, USA) in accordance with the manufacturer’s instructions. Experiments were conducted after 48 hours of gene silencing.

### Western blotting analysis

Cell lysates were obtained using LDS sample buffer (NuPAGE; Invitrogen Corp., Carlsbad, CA, USA). Proteins were resolved on Tris-glycine gels and transferred onto PVDF membranes. Blots were probed with commercial primary antibodies and enhanced chemiluminescence detection using (ECL-Plus; Amersham Biosciences Inc., Piscataway, NJ, USA). Antibodies used were: PARP1 (#9542), p21 (#2947), Puma (#4976), TFIIB (#4169), phospho-p53 (Ser20) (#9287), phospho-p53 (Ser46) (#2521) and acetyl-p53 (Lys382) (all Cell Signaling Technology/(Danvers, MA, USA); acetyl-p53 (Lys120; #BAM-71-131-EX; Cosmo Bio, Yokohama, Japan); p53 (#sc-126), Mdm2 (#sc-965) and Bax (#sc-493) (all from Santa Cruz Biotechnology, Santa Cruz, CA, USA); phospho-histone H2A.X (#06-570; Millipore Corp., Billerica, MA, USA). Anti-actin (#A5316; (Sigma-Aldrich) and anti-tubulin (#2148; Cell Signaling Technology) were used as loading controls.

### Real-time quantitative PCR

Total cellular RNA was isolated from U-2 OS cells (RNeasy Mini Kit; Qiagen) in accordance with the manufacturer’s instructions. A high-capacity cDNA reverse transcription kit (Applied Biosystems, Foster City, CA, USA) was used to reverse-transcribe RNA into cDNA, and a SYBR Green system (Fast SYBR Green Master Mix; Applied Biosystems) was used for DNA amplification on a PCR system (7500 Fast Real-Time PCR System; Applied Biosystems). The data were analyzed by Sequence Detection Systems Software (version 2.3). For the primer sequences for the respective genes, see Additional file 1: Table S1.

### Subcellular fractionation

U-2 OS cells were treated with trypsin, rinsed twice with PBS, and then lysed at 4°C with isotonic buffer (10 mmol/l Tris HCl, pH 7.5, 2 mmol/l MgCl_2_, 3 mmol/l CaCl_2_, 3 mol/l sucrose) supplemented with 1 mmol/ldithiothreitol (DTT) and protease inhibitor cocktail (set III; Calbiochem, San Diego, CA, USA). Lysis buffer was used at 100 μl per 20 μl of packed cell volume (PCV). Detergent (IGEPAL CA-630; Rhodia, La Défense, France) was then added to a final concentration of 0.03 to 0.05%, and sample was immediately separated by centrifugation for 30 seconds at 11,000 g and 4°C. The resulting supernatant was taken as the cytoplasmic fraction. The pellet was then rinsed twice with 100 μl isotonic buffer, and taken as the nuclear fraction for western blot analysis.

### Time-lapse microscopy and image analysis

Cells were plated in 35 mm imaging dishes (μ-dish; ibidi, Planegg, Germany) and cultured in phenol red-free CO_2_-independent medium (Invitrogen) supplemented with 10% FCS, 100 U/ml penicillin, and 100 μl streptomycin. Cell images were acquired with an inverted microscope (TE2000-PFS; Nikon, Tokyo, Japan) enclosed in a humidified chamber maintained at 37°C. Cells were imaged every 10 minutes using a motorized stage and a ×20 objective.

For data plotted in the cumulative survival curves and the phenotype distributions, we viewed and analyzed the images manually by eye, using MetaMorph software (Molecular Dynamics Inc., Sunnyvale, CA, USA). In the phase-contrast images, we scored by morphological tracking the following: interphase (by flat morphology), entry into mitosis (by cell rounding), cell division (by re-spreading and splitting), cell-cycle arrest (by absence of cell division for 72 hours) and cell death (by blebbing followed by cell lysis). For quantifying the single-cell p53 traces, we used an automatic cell-tracking program that we developed using MatLab. The program consists of image-analysis procedures that sequentially segment the individual cells, track them in time, identify the nucleus, and measure the p53 fluorescence intensity in the nucleus and cytoplasm, respectively. Based on the quantified p53 fluorescent trajectories, we classified the dynamic mode of nuclear p53 manually by eye. We categorized as ‘oscillatory’ any single-cell p53 signals in the nucleus that exhibited periodic fluctuations in intensity between a high fluorescent state and a low state near background fluorescence. We defined a nuclear p53 signal that monotonically rose to and sustained at a high fluorescence level as ‘monotonic increase’. Most (approximately 95%) of the p53 trajectories had obvious features that could be unambiguously categorized into the two distinct modes. For the 5% of trajectories that had ambiguous features (mostly occurring at the intermediate DNA damage level), we discarded these trajectories from further analysis.

## Competing interests

All authors declare that they have no competing interests.

## Authors’ contributions

XC performed experiments, analyzed data and wrote the paper; JC and SG developed analytical tools and analyzed data; HG and YZ performed experiments; QO designed the study and wrote the paper; JS designed the study, designed the experiments, analyzed data, and wrote the paper. All authors read and approved the final manuscript.

## Supplementary Material

Additional file 1 **Supplementary Information.** This PDF file contains the supplementary Figure S1 to S4 and Supplementary Table S1. Click here for file
