# Supplementary material for: DNA damage strength modulates a bimodal switch of p53 dynamics for cell-fate control
Source: BMC Biol. 2013 Jun 21;11:73. doi: 10.1186/1741-7007-11-73 (PMC3702437; doi:10.1186/1741-7007-11-73)
Supplement: Additional file 1 — Supplementary Information. This PDF file contains the supplementary Figure S1 to S4 and Supplementary Table S1. [file 1741-7007-11-73-S1.pdf]

DNA damage strength modulates a bimodal switch of p53 dynamics for cell fate control

Xi Chen[1], Jia Chen[2], Siting Gan[2], Huaji Guan[1], Yuan Zhou[1], Qi Ouyang[2,*] and Jue Shi[1,*]

[1]Center for Quantitative Systems Biology and Department of Physics, Hong Kong Baptist University, Hong Kong, China

[2]Center for Quantitative Biology and State Key Laboratory for Mesoscopic Physics, Peking University, Beijing, China

Supplementary Information

Supplementary Figures

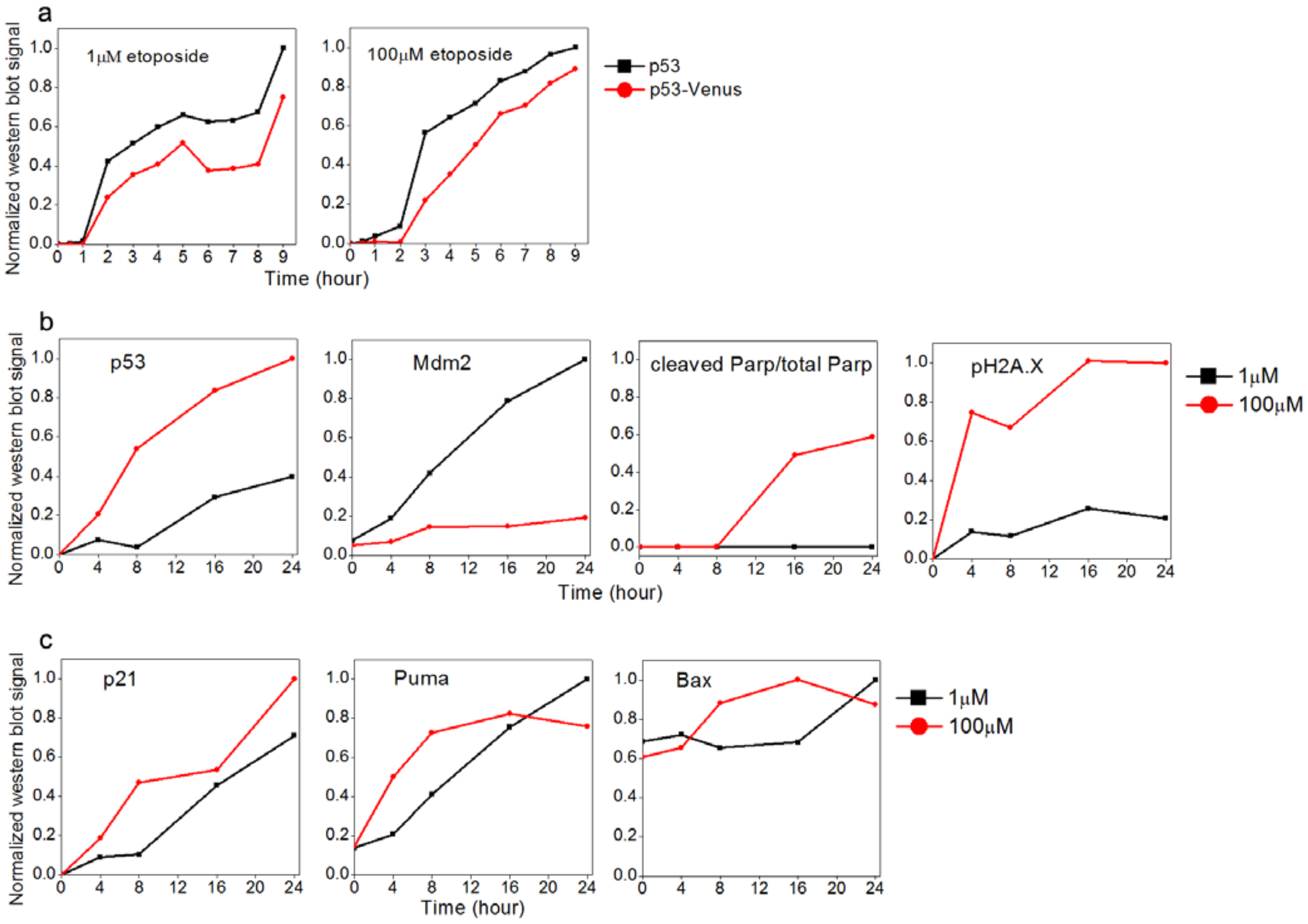

Supplementary Figure S1. Protein dynamics quantified from western blots shown in Fig. 1b (a), Fig. 3b (b) and Fig. 4b (c), respectively. In all graphs, the western blot signals at different time points were normalized to the maximal signal at the final time point.

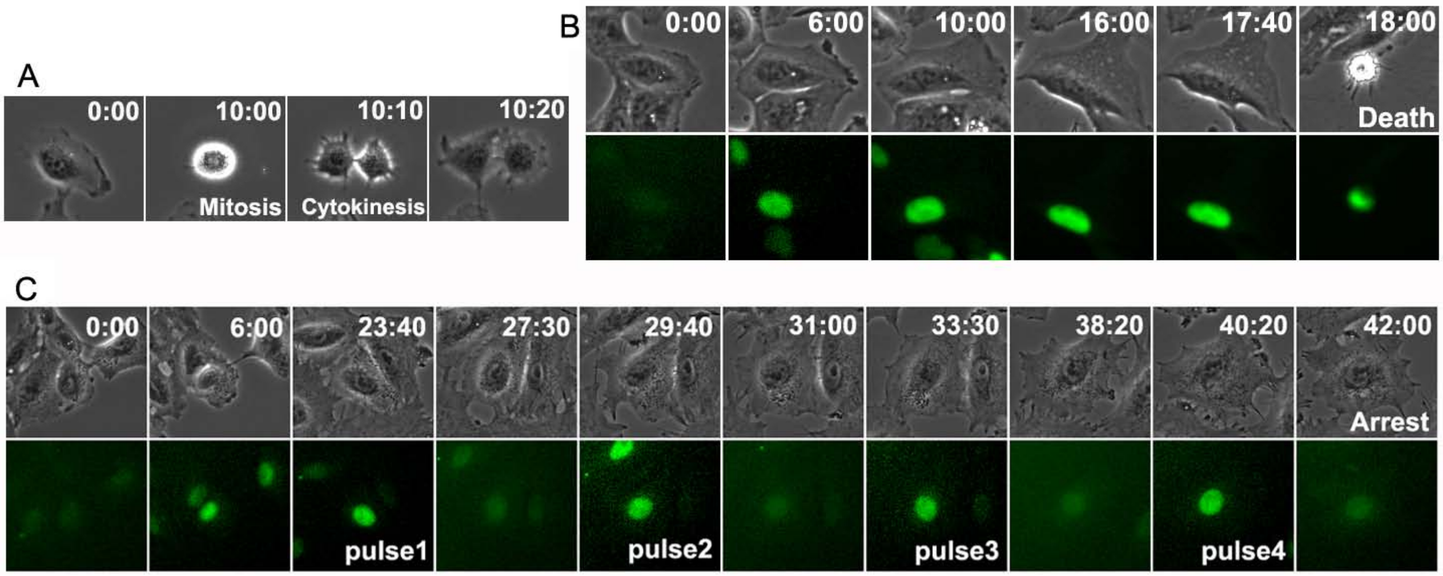

Supplementary Figure S2. Still images of representative U-2 OS cells from time-lapse movies. (a) a normally dividing cell without drug; (b) a dead cell under 100μM etoposide; (c) a cell in cell-cycle arrest under 1μM etoposide. Upper panel: phase-contrast images (elapse time shown in hour:minute). Lower panel: corresponding fluorescence of the p53-Venus reporter shown in green.

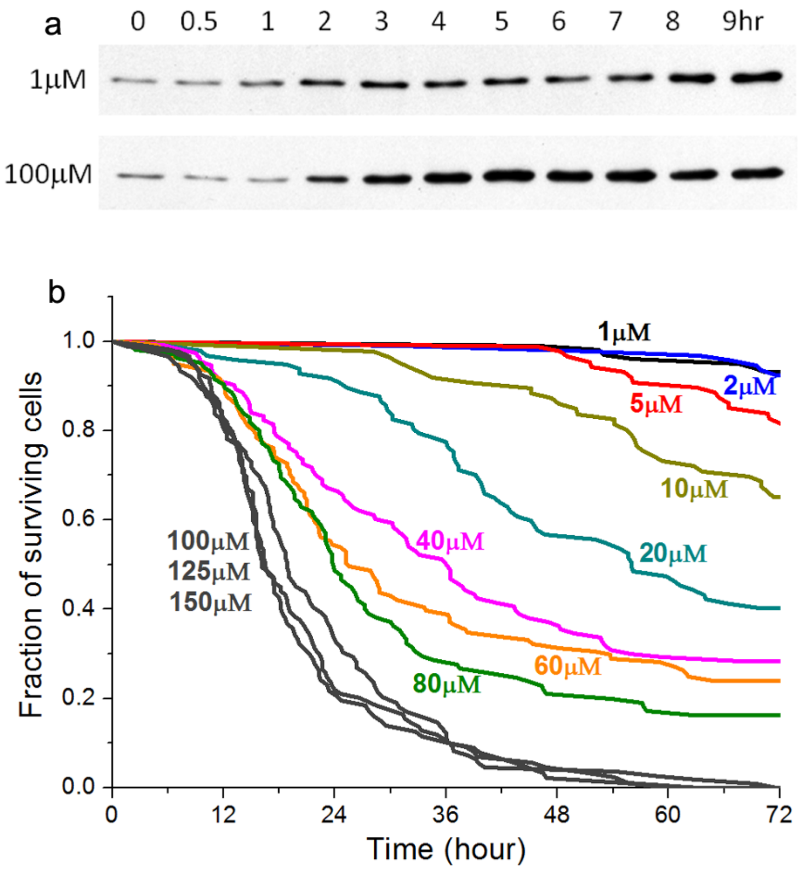

Supplementary Figure S3. Dose response of the parental U-2 OS cell line to etoposide.

(a) Induction dynamics of p53 at low (1μM) and high (100μM) etoposide concentration.

(b) Cumulative survival statistics at the indicated etoposide concentrations. Total number of cells analyzed for each curve ranges from 86 to 121, varied between conditions. Individual cells were monitored by phase-contrast and fluorescence time-lapse microscopy from drug addition for 72 hrs or till morphological death occurred. Kinetics of cell death was plotted as cumulative survival curves.

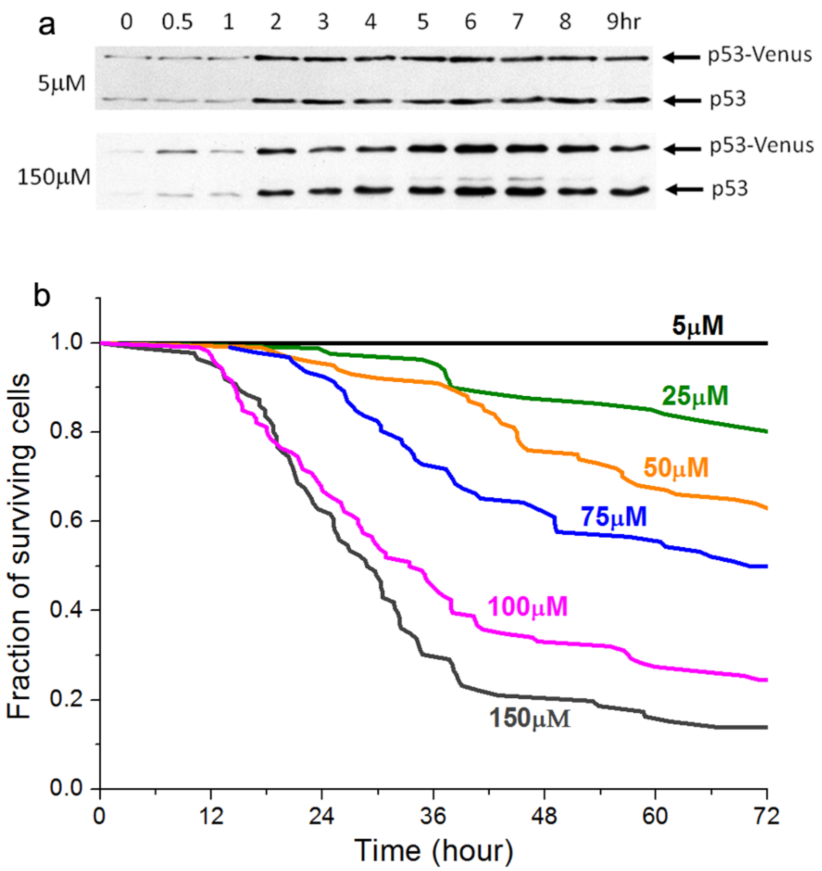

Supplementary Figure S4. Dose response of the A549-p53 reporter line to etoposide.

(a) Induction dynamics of p53 at low (5μM) and high (150μM) etoposide concentration.

(b) Cumulative survival statistics at the indicated etoposide concentrations. Total number of cells analyzed for each curve ranges from 81 to 106, varied between conditions.

Supplementary Table S1: Primer sequences for the real-time RT-PCR experiments.

Primer Name	Sequence (5' to 3')
P21 forward	TGAGCCGCGACTGTGATG
P21 reverse	GTCTCGGTGACAAAGTCGAAGTT
GAPDH forward	TATTGGGCGCCTGGTCACCA
GAPDH reverse	CCACCTTCTTGATGTCATCA
GADD45A forward	CCATGCAGGAAGGAAAACTATG
GADD45A reverse	CCCAAACTATGGCTGCACACT
XPC forward	CCCAGCCCGCTTTACCA
XPC reverse	TGCATTAACTGTAAATGTTCCAATGA
WIP1 forward	TAGTGGTGCTCAGCCTGCAA
WIP1 reverse	TCTGCGTCGCATGGTGAGT
BAX forward	CTGAGCTGACCTTGGAGC
BAX reverse	GACTCCAGCCACAAAGATG
APAF1 forward	CACGTTCAAAGGTGGCTGAT
APAF1 reverse	TGGTCAACTGCAAGGACCAT
TP53AIP1 forward	CCAAGTTCTCTGCTTTC
TP53AIP1 reverse	AGCTGAGCTCAAATGCTGAC
PML forward	CGGAGGAGGAGTTCCAGTTT
PML reverse	CCACAATCTGCCGGTACAC
PUMA forward	GCGAGACTGTGGCCTTGTGT
PUMA reverse	CGTTCCAGGGTCCACAAAGT
NOXA forward	TGGAAGTCGAGTGTGCTACTCAA
NOXA reverse	CAGAAGAGTTTGGATATCAGATTCAGA

The primers for gene YPEL3 were purchased from Qiagen (#PPH15441A-200)
